# Study on 3D Effects on Small Time Delay Integration Image Sensor Pixels

**DOI:** 10.3390/s25071953

**Published:** 2025-03-21

**Authors:** Siyu Guo, Quan Zhou, Pierre Boulenc, Alexander V. Klekachev, Xinyang Wang, Assaf Lahav

**Affiliations:** 1 Changchun Institute of Optics, Fine Mechanics and Physics, Chinese Academy of Sciences, Changchun 130033, China; siyu.guo@gpixel.com; 2 University of Chinese Academy of Sciences, Beijing 100049, China; 3 Gpixel Inc., Changchun 130031, China; quan.zhou@gpixel.com (Q.Z.); pierre.boulenc@gpixel.com (P.B.); alexander.klekachev@gpixel.com (A.V.K.); xinyang.wang@gpixel.com (X.W.)

**Keywords:** CCD-in-CMOS, TDI image sensor, 3D effect, TCAD simulation

## Abstract

This paper demonstrates the impact of 3D effects on performance parameters in small-sized Time Delay Integration (TDI) image sensor pixels. In this paper, 2D and 3D simulation models of 3.5 μm × 3.5 μm small-sized TDI pixels were constructed, utilizing a three-phase pixel structure integrated with a lateral anti-blooming structure. The simulation experiments reveal the limitations of traditional 2D pixel simulation models by comparing the 2D and 3D structure simulation results. This research validates the influence of the 3D effects on the barrier height of the anti-blooming structure and the full well potential and proposes methods to optimize the full well potential and the operating voltage of the anti-blooming structure. To verify the simulation results, test chips with pixel sizes of 3.5 μm × 3.5 μm and 7.0 μm × 7.0 μm were designed and manufactured based on a 90 nm CCD-in-CMOS process. The measurement results of the test chips matched the simulation data closely and demonstrated excellent performance: the 3.5 μm × 3.5 μm pixel achieved a full well capacity of 9 ke- while maintaining a charge transfer efficiency of over 0.99998.

## 1. Introduction

TDI image sensors exhibit enhanced light sensitivity and an improved signal-to-noise ratio, making them particularly effective for low-light and high-frequency working conditions. TDI image sensors are widely utilized in various applications, including space remote sensing, aerial imaging, industrial monitoring, and security surveillance [1]. Charge-Coupled Devices (CCDs) remain predominant in TDI image sensors operating in the charge domain. However, the special manufacturing processes associated with CCDs have restricted their accessibility for academic institutions and enterprises that aim to assess and implement CCD technology for innovative applications [2].

The advancement of CMOS technology has facilitated the development of digital domain TDI image sensors that utilize CMOS pixels, which have an advantage in enhanced chip integration and reduced readout noise [3,4]. Nevertheless, the implementation of digital domain superposition in TDI imaging sensors needs large-scale Analog-to-Digital Converter (ADC) arrays, which could result in significant increases in chip size and heightened complexity in both peripheral circuitry and algorithmic frameworks [5,6,7,8]. A notable recent advancement is the introduction of monolithic CCD-in-CMOS technology [9,10,11], wherein the charge collection and transmission architecture of the CCD is manufactured through advanced CMOS processes [12,13,14]. Furthermore, complex image signal acquisition and quantization circuit functions are integrated into the chip through CMOS processing, effectively enhancing image quality [15,16,17,18,19,20].

In semiconductor wafer inspection, high-throughput gene sequencing, and biological fluorescence imaging, small-sized TDI image sensors are widely used due to the higher resolution and Modulation Transfer Function (MTF) performance requirements of these applications. In the small-sized TDI pixels design, 2D simulation models are commonly employed for rapid simulation and verification of pixel performance parameters. However, the presence of 3D effects within the actual pixel architecture frequently results in discrepancies between the performance parameters obtained from 2D simulation models and the data from real tests.

This paper demonstrates the distinctions between the 3D small-sized pixel simulation model and the 2D simulation model in terms of effective threshold voltage and the height of the anti-blooming barrier. These two parameters determine the pixel’s Full Well Capacity (FWC) and anti-blooming capability, which directly influence the imaging quality of the sensor. In order to validate the simulation data, test chips with pixel sizes of 3.5 μm × 3.5 μm and 7.0 μm × 7.0 μm were designed and manufactured based on an advanced 90 nm CCD-in-CMOS process. The measurement results of the test chips matched the 3D simulation data closely and demonstrated high performance: the 3.5 μm × 3.5 μm pixel achieved 9 ke- full well capacity while maintaining over 0.99998 Charge Transfer Efficiency (CTE).

This paper is organized as follows: Section 2 introduces the $V_{EFF}$ model in a buried channel and explains the working principle of the lateral anti-blooming structure. Section 3 shows the influence of 3D effect on pixel full well potential and anti-blooming capability performance. Section 4 presents the test chips’ FWC and anti-blooming capability test results before and after pixel design optimizations based on the simulation results, which are followed by conclusions and comparisons with other TDI imaging sensors in Section 5.

## 2. Introduction of Pixel Full Well Potential and Lateral Anti-Blooming Structure

**Figure 1:**
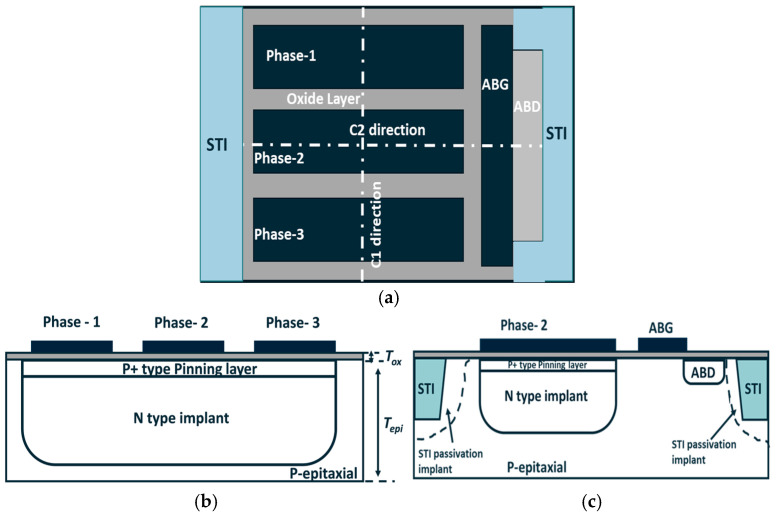
(**a**) Schematic of 3-phase pixel unit (top view). (**b**,**c**) Cross section of 3-phase pixel along C1 and C2 directions in (**a**).

Figure 1 depicts a schematic of a three-phase pixel. As illustrated in Figure 1, the pixel utilizes a buried channel design and integrates a lateral anti-blooming structure, comprising an anti-blooming gate (ABG) and anti-blooming drain (ABD) [15] to control the over-saturation under strong light conditions. In Section 2, we will provide a detailed analysis of the potential profile in the buried channel under different gate voltages based on the schematic structure presented in Figure 1.

**Figure 2:**
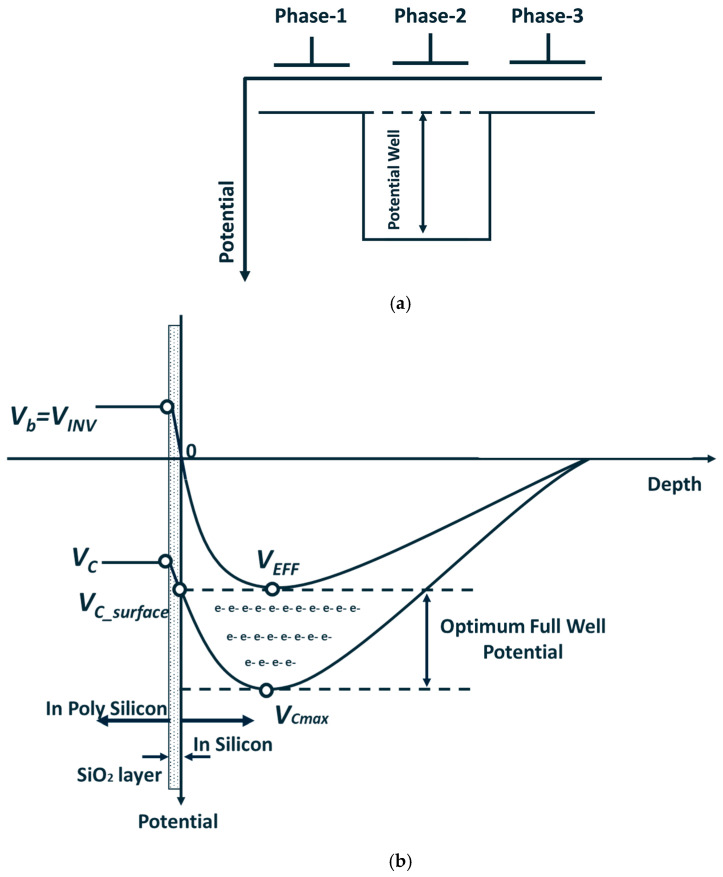
(**a**) Potential profile in buried channel from phase-1 to phase-3. (**b**) Potential profile in buried channel vertical to phase-2 and phase-1.

Referring to Figure 1, assuming a negative gate voltage is applied to phase-1 and phase-3, while a positive gate voltage is applied to phase-2, the potential profile along the buried channel from the phase-1 to phase-3 direction is illustrated in Figure 2. Under this condition, a potential well forms beneath phase 2, while the channel potential at phases 1 and 3 functions as barriers surrounding this well. Consequently, phase-2 is classified as the collection phase, while phase-1 and phase-3 are classified as barrier phases.

The maximum channel potential ($V_{Cmax}$) and potential at the SiO_2_-Si interface ($V_{C\_surface}$) will be lower with a reduction in gate voltage. However, due to the presence of the P-type pinning layer, the surface potential at the SiO_2_-Si interface remains pinned (i.e., $V_{surface}=V_{Epi}=0\;V$) at 0 V, which is called the pinning condition. Under this condition, reducing the gate voltage does not affect the potential distribution within the buried channel, as the reduction in gate voltage is applied to the oxide layer. Under the pinning condition, the maximum potential under the CCD phase is called the effective threshold voltage ($V_{EFF}$), as shown in Figure 2b. The difference between $V_{EFF}$ and $V_{Cmax}$ is defined as the full well potential. When $V_{C\_surface}=V_{EFF}$, as depicted in Figure 2b, the full well potential will be called the optimum full well potential. The only method to change the $V_{EFF}$ is to adjust the buried channel conditions. For example, decreasing the N-type implant of the buried channel will lower $V_{EFF}$ under the barrier phase. However, the maximum potential ($V_{C\_max}$) also will be lowered simultaneously. Considering that the optimum full well potential ($V_{OFW}$) is equal to the difference between $V_{C\_max}$ and $V_{EFF}$, under the lower N-type implant buried channel process condition, the gate voltage of the collection phase will also be decreased to achieve optimum full well conditions, which will cause lower $V_{OFW}$ values. Correspondingly, the higher $V_{EFF}$ value can increase the full well potential, consequently increasing the pixel full well capacity [21]. This approach serves as the primary strategy for enhancing full well performance in small-size pixel design.

**Figure 3:**
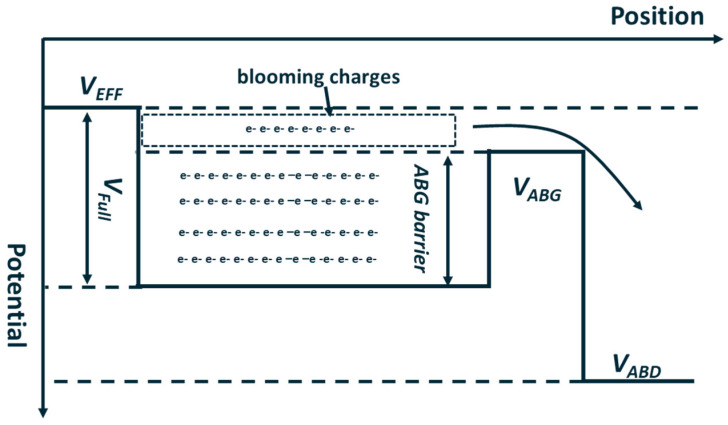
Schematic of horizontal anti-blooming structure potential profile.

Severe pixel-to-pixel blooming can significantly degrade the imaging quality of image sensors. To mitigate the blooming phenomenon, the anti-blooming structure is utilized in pixel design. Figure 3 illustrates a schematic of the lateral anti-blooming structure, which comprises an ABG and a heavily N-type implant ABD. For the ABG to operate effectively, the potential under the anti-blooming gate needs to be set higher than $V_{EFF}$. When the accumulated charges are sufficient to lower the collection phase’s maximum channel potential below the ABG potential, the excess charges (i.e., blooming charges) will overflow to the ABD due to the potential difference between the potential well and ABG.

The introduction of anti-blooming structures optimizes the performance of TDI pixels under strong light, but it also brings some challenges in pixel design, particularly for smaller pixels. In these smaller pixels, the anti-blooming structure can significantly impact the full well capacity and charge collection efficiency. Therefore, optimizing pixel performance by building simulation models is a crucial step in small-sized TDI pixel design. Two-dimensional models are extensively utilized in pixel simulation due to their advantages in terms of simulation speed and efficiency. However, the performance data obtained from 2D simulations frequently show significant differences when compared to the actual test data. The presence of 3D effects in pixel structures leads to inaccuracies in the pixel performance parameters obtained from 2D simulation models, resulting in discrepancies with the actual test data of the pixels. This study will provide a comprehensive analysis of the limitations of the 2D simulation model and the reasons for these differences between simulation and test data in the following sections.

## 3. Three-Dimensional Effect’s Influence on Pixel Full Well Potential and Anti-Blooming Capability Performance Simulation

**Figure 4:**
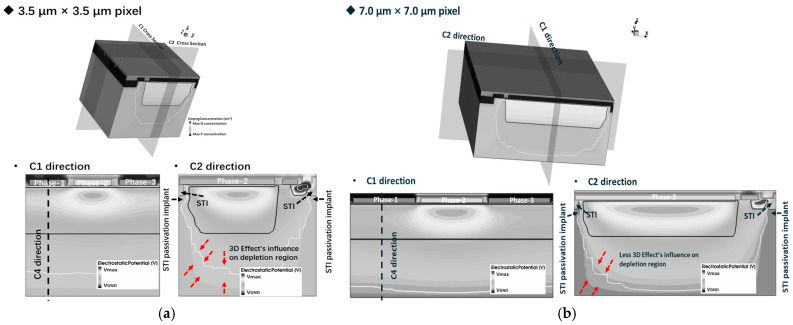
(**a**) Three-dimensional simulation model of 3.5 μm × 3.5 μm 3-phase pixel with lateral AB structure. (**b**) Three-dimensional simulation model of 7.0 μm × 7.0 μm 3-phase pixel with lateral AB structure.

To investigate the influence of the 3D effect on pixel full well potential and anti-blooming capability, based on a 90 nm CCD-in-CMOS process, we construct a 3D Technology Computer-Aided Design (TCAD) simulation model of a 3.5 μm × 3.5 μm three-phase pixel with a lateral anti-blooming structure, as illustrated in Figure 4. The oxide layer is thinner than 10 nm, the epitaxial layer is 2.3 μm, and the depletion depth of the buried channel is about 2.0 μm. Additionally, Figure 4b depicts a 7.0 μm × 7.0 μm three-phase pixel 3D simulation structure. All process conditions (including species, dose, energy, tilt, and rotation angle) in different size simulation structures are same. The primary distinction lies in the CCD phase size of the 7.0 μm × 7.0 μm pixel, which is 5.2 times larger than that of the 3.5 μm × 3.5 μm pixel.

**Figure 5:**
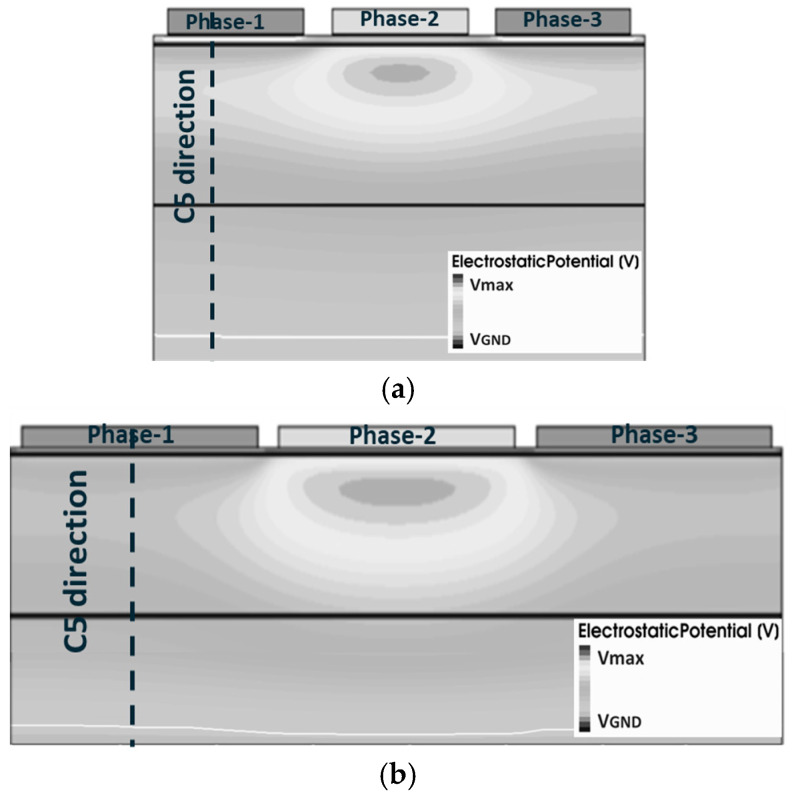
(**a**) 3.5 μm × 3.5 μm pixel 2D simulation structure along phase-1 to phase-3 direction; (**b**) 7.0 μm × 7.0 μm pixel along the same direction.

**Figure 6:**
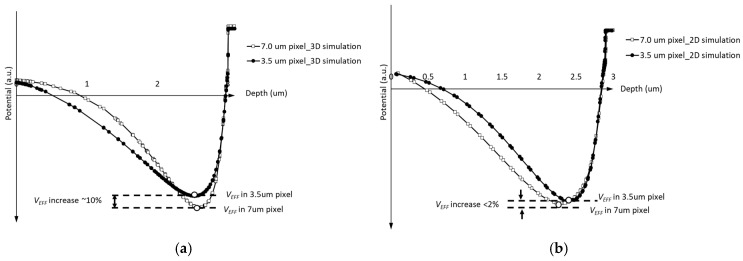
(**a**) $V_{EFF}$ comparison between 3.5 μm × 3.5 μm and 7.0 μm × 7.0 μm pixels from 3D simulation structure. The $V_{EFF}$ comparison from the 2D simulation structure is shown in (**b**).

The 2D simulation structure of the 3.5 μm × 3.5 μm and 7.0 μm × 7.0 μm pixel C1 direction is illustrated in Figure 5a,b. Figure 6a,b present a comparative analysis of the 2D and 3D simulation structures on $V_{EFF}$ for pixel dimensions of 3.5 μm × 3.5 μm and 7.0 μm × 7.0 μm, respectively.

Setting phase-2 as the barrier phase ($V_{b}<0\;V$), Figure 6a shows the potential profile along the C4 direction from Figure 4a,b. From Figure 6a, it is observed that the $V_{EFF}$ varies with pixel size. The $V_{EFF}$ value from the 7.0 μm × 7.0 μm pixel simulation structure is approximately 0.2 V higher than that in the 3.5 μm × 3.5 μm simulation. For pixel isolation between different columns, the Shallow Trench Isolation (STI) structure is implemented. Due to the presence of STI surface traps, an additional p-well implant is added around the STI, which is known as an STI passivation implant. The lateral diffusion of the STI passivation implant will influence the buried channel profile, especially in small-size pixels with shorter CCD gate lengths. Compared with the 3.5 µm pixel, the extended gate length of the 7.0 µm pixel CCD gate results in a reduced influence of the STI passivation implant on the buried channel doping profile. Consequently, as depicted in the curve presented in Figure 6a, the 7 µm pixel shows a higher $V_{EFF}$ value.

The potential profile obtained from the 2D simulation structure along the C5 direction is illustrated in Figure 6b. As observed in Figure 6b, the $V_{EFF}$ difference between the 3.5 μm and 7.0 μm pixel is smaller than that in Figure 6a. This discrepancy arises because the 2D simulation only considers the implant influence at the current cross-section on the buried channel. The distribution of the STI passivation implant does not align with the plane of the cross-section depicted in Figure 5; therefore, it is impossible to demonstrate the effect of the STI passivation implant on $V_{EFF}$. Analysis of the simulation results presented in Figure 6 demonstrates that the $V_{EFF}$ value of the pixel is affected by the distribution of implants across different directions. This finding suggests the presence of a 3D effect in pixel simulations, thereby indicating that the utilization of the 2D simulation model to evaluate the performance parameters of the pixel may yield inaccurate results.

**Figure 7:**
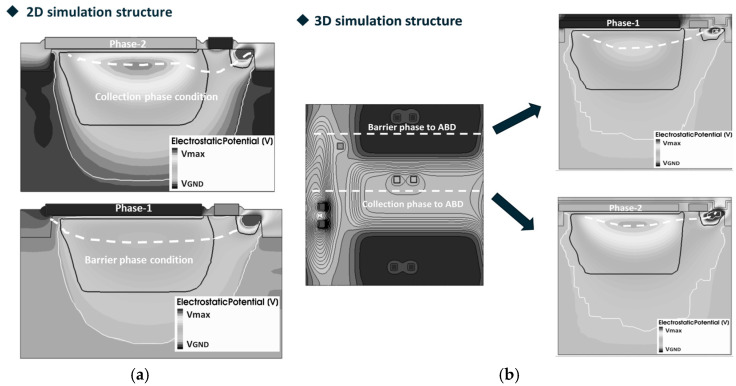
(**a**) 3.5 μm × 3.5 μm pixel 2D simulation structure used to simulate the ABG barrier; (**b**) 3.5 μm pixel 3D simulation structure used to simulate the ABG barrier. The white dashed lines in (**a**) and the C7 and C8 direction in (**b**) represent the position of the maximum potential distribution in the epitaxial layer.

When designing pixels for TDI image sensors, it is crucial to validate the operation range of the anti-blooming structure through Technology Computer-Aided Design (TCAD) simulation. This process helps to determine whether optimization of the ABG’s process parameters is necessary. Figure 7a,b illustrate the potential profile from the CCD gate to the ABD under the barrier phase and collection phase from the 3.5 μm pixel 2D and 3D simulation structures, respectively.

**Figure 8:**
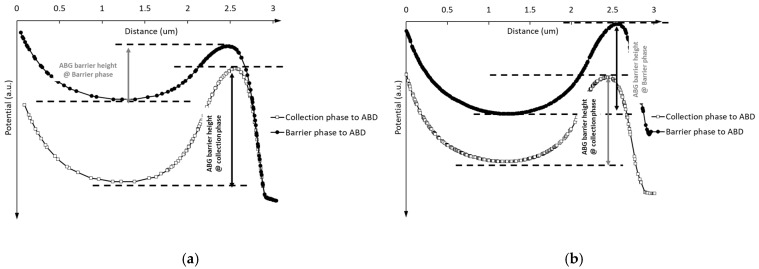
ABG barrier and the potential profile from the CCD gate to the ABD under the barrier phase condition and collection phase from (**a**) 2D simulation and (**b**) 3D simulations.

The potential distribution from the barrier phase and collection phase to the ABD under the 2D and 3D simulation structure is shown in Figure 8.

Compared with Figure 8a,b, it is obvious that the ABG barrier height, when the CCD gate is set as the barrier phase, differs significantly between the 2D and 3D simulations. Notably, the barrier height in the 3D simulations remains nearly constant, regardless of whether the CCD phase is set in the barrier or collection phase. The simulation results in Figure 8a,b further demonstrate the limitations of 2D simulation structures in pixel design. The 2D simulation structure in Figure 7a represents a cross-section of the 3D simulation structure in Figure 4 at a specific position along the phase-2-to-ABD direction. The data obtained from Figure 8a can only characterize the barrier height from a specific position in phase-2 to the ABG. It is important to note that the barrier height near phase-2 does not accurately represent the barrier height near phase-1 or phase-3 under the barrier phase conditions.

**Figure 9:**
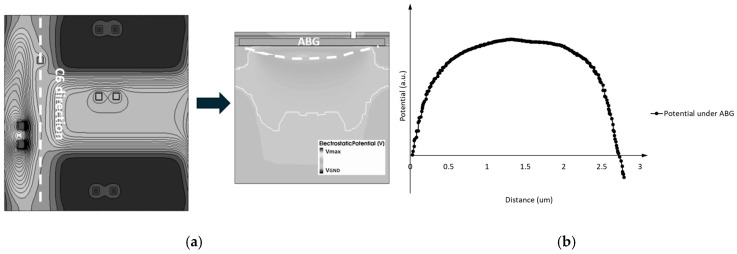
(**a**) Top view of the electric potential distribution of the 3D pixel simulation structure and potential distribution under ABG; (**b**) maximum potential profile under the ABG.

Figure 9a presents a top view of the electric potential distribution in the 3D pixel simulation structure. From Figure 9a, it can be observed that the implant profile of the ABD on the left side of the ABG is not uniformly distributed along the ABG. The electric potential distribution of the ABG shows a pattern of lower potential at both ends and a higher potential in the middle, as illustrated in Figure 9b. Due to the presence of 3D effects, the electric potential distribution beneath the ABG is influenced not only by the gate voltage of the ABG but also by the potential distribution conditions on both sides. The influence of 3D effects on ABG potential also suggests a method to adjust the gate voltage working range of the ABG: by introducing an additional implant near the ABG, the doping distribution around the ABG will be changed, thereby achieving the aim of adjusting the barrier height.

The aforementioned simulation results not only demonstrate the limitations of the conventional 2D structure for small-pixel simulations but also confirm the presence of 3D effects within the pixels and their impact on the pixel full well potential and ABG barrier. To validate the simulation results in this section, test chips were fabricated based on the advanced 90 nm CCD-in-CMOS process described the next section.

## 4. Pixel Performance Evaluation of the Test Chip

**Figure 10:**
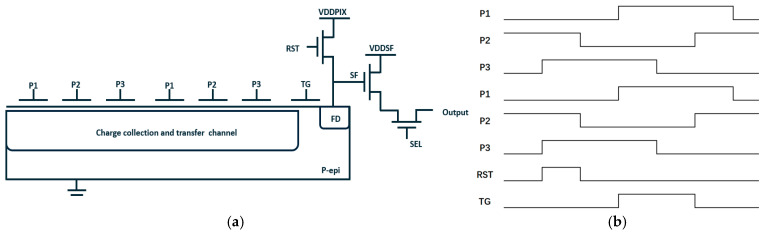
(**a**) Schematic of the designed pixels’ structure; (**b**) working timing in the imaging sensor’s performance test.

**Figure 11:**
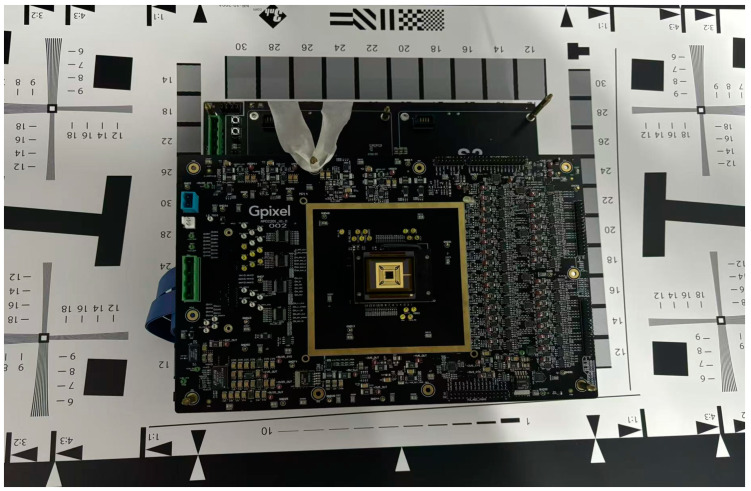
Photo of the test chip and its evaluation board.

In order to validate the simulation results, a series of 3.5 μm × 3.5 μm and 7.0 μm × 7.0 μm test chips based on the standard 90 nm CCD-in-CMOS process were manufactured. A schematic of the TDI image sensors and their 3-phase working timing is shown in Figure 10a,b, and Figure 11 shows the shape of test chip and its evaluation board.

**Figure 12:**
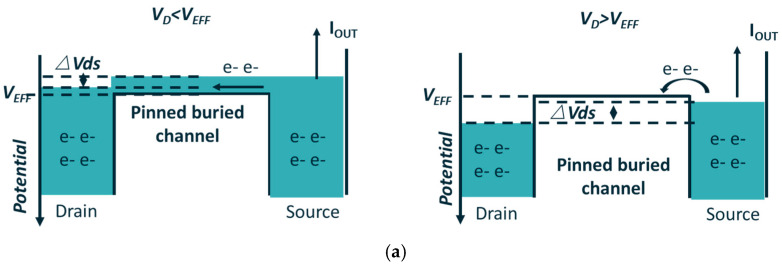

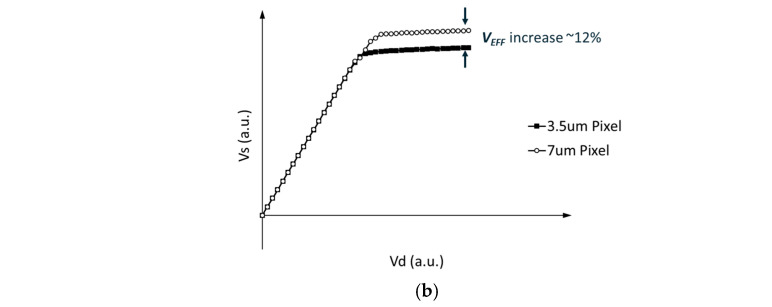
(**a**) Schematic of the FS method with JEFT structure; (**b**) PCM test results of $V_{EFF}$ from Fab. The inflection point of the *V_s_*–*V_D_* curve is the measured $V_{EFF}$ according to the floating source method in [23].

The process conditions for the 3.5 μm × 3.5 μm and 7.0 μm × 7.0 μm test chips were consistent with the 3D simulation in Section 3. Based on the JEFT structure used to test $V_{pin}$ in PPD [22], and with the assistance of the Process Control Monitor (PCM) structures, we optimized the floating source method to measure $V_{EFF}$. The Floating Source (FS) method [23] involves leaving the source of a JFET structure floating and monitoring its source potential ($V_{s}$) as a function of the biasing voltage applied to the drain ($V_{d}$). During the measurement, a current $I_{out}$ is forced at the source, as shown in Figure 12a. The $V_{EFF}$ test results are shown in Figure 12b.

The comparisons of $V_{EFF}$ between the 3.5 μm × 3.5 μm and 7.0 μm × 7.0 μm pixels based on the PCM test results for the 2D and 3D simulation models are shown in Table 1. All data in Table 1 were normalized based on the 3D simulation results of the 3.5 μm pixel. Based on Table 1, the $V_{EFF}$ value from the 3D simulation model matches the PCM test result better than that from the 2D simulation model. The PCM test result closely matches the 3D simulation result.

**Figure 13:**
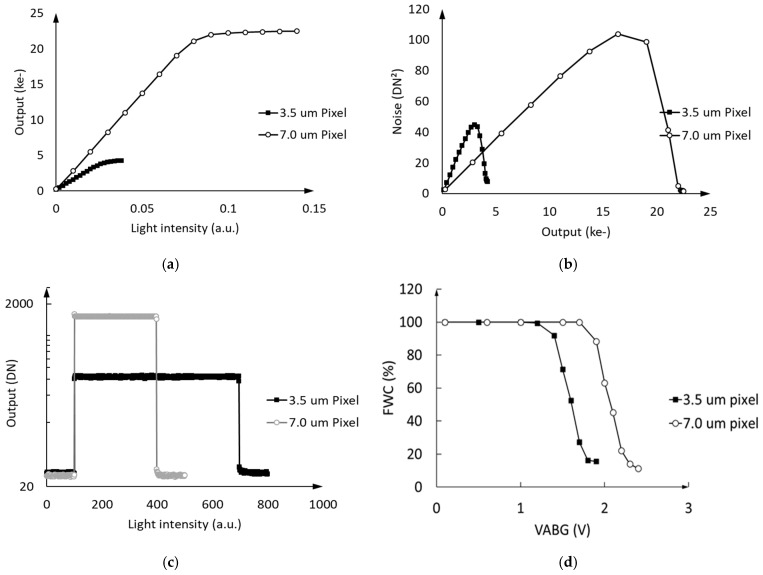
The response (**a**), photon transfer (**b**), and CTE curve (**c**) tested. The relationship between the ratio of FWC and $V_{ABG}$ is shown in (**d**).

The response and photon transfer curves tested under the timing in Figure 10b are shown in Figure 13a,b. All the data were tested under the optimum full well condition. The FWC at the top of Photon Transfer Curve (PTC) under the 3.5 μm pixel condition was ~3 ke-, and the FWC at the top of the PTC under the 7.0 μm pixel condition was ~16 ke-. Since the full well potential is proportional to $V_{EFF}$, the well capacity of a pixel can be approximately estimated as the product of the well capacitance and the full well potential. The changes in full well capacity measured in Figure 13 match the 3D simulation results. Consequently, the test data in Figure 13 also verify the existence of the 3D effect in the pixels.

The comparisons of FWC between the 3.5 μm × 3.5 μm and 7.0 μm × 7.0 μm pixels from the 3D simulation model and chip measurement are shown in Table 2. All the data in Table 2 were normalized based on the 3D simulation results of the 3.5 μm pixel.

All of test chips’ CTE curves in Figure 13c were tested under the three-phase working model using the Extended Pixel Edge Response (EPER) method [24,25]. The CTE performances of the 3.5 μm × 3.5 μm and 7.0 μm × 7.0 μm test chips were higher than 0.99998. To verify the working range of the ABG, the relationship between the ratio of FWC and $V_{ABG}$ is shown in Figure 13d.

**Figure 14:**
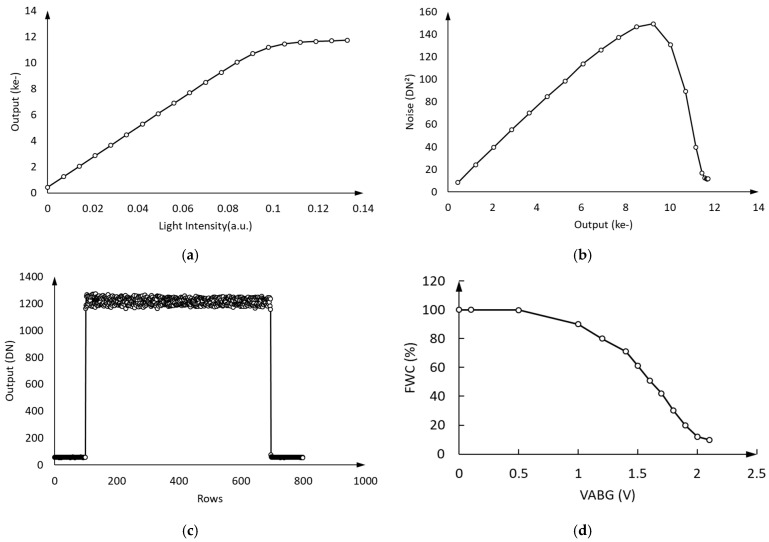
After process optimization, the response (**a**), photon transfer (**b**), and CTE curve (**c**). The relationship between the ratio of FWC and $V_{ABG}$ is shown in (**d**).

In Figure 14, we improved the process parameters of the pixels by optimizing the effective threshold voltage of the pixel buried channel and potential distribution under the ABG. The 3.5 μm × 3.5 μm pixel achieved a full well capacity of 9 ke- while maintaining a CTE of over 0.99998, and the ABG could work in wider voltage range (the ABG started to work at 0.5 V).

Generally, a significant increase in full well capacity will increase the possibility of a decrease in CTE performance. If the increase in full well capacity causes a decrease in CTE (for example, CTE < 0.9999), the lower CTE will cause degradation in image quality (there will be some tailing in the image), making the optimization of full well capacity ineffective.

For scientific TDI image sensors, a CTE of above 0.99995 is required. According to the test results in Figure 14, the CTE performance remains consistent (greater than 0.99998) before and after the optimization of the full well capacity, indicating that the optimization of the full well capacity (from 3 ke- to 9 ke-) is indeed effective.

## 5. Summary and Discussion

Through a comparative analysis of the full well potential and ABG barrier height between the 2D and 3D simulation structures, this study not only demonstrates the existence of 3D effects but also discusses the limitations of 2D simulation structures. While the utilization of 2D simulation structures has the advantage of efficiently obtaining simulation results, constructing 3D models is essential for acquiring more accurate pixel performance data.

For small-sized pixels such as the one in this paper with a pitch lower than 3.5 μm, it is necessary to use 3D models to simulate the performance parameters. For large-size pixels, such as those with a pixel pitch larger than 7 μm, 3D simulation models will take too much time to build (whereas building a 14 μm pixel model will require more than 2 weeks). It is more efficient to split the device into several components to build 2D or 3D models of each part. In many cases, 2D simulations are commonly used for qualitative studies of devices, while 3D models are often used in quantitative research to obtain more accurate data. The decision of whether to use a 3D simulation model depends on the specific parameters and requirements of the research.

Following the validation of 3D effects through simulation, this project utilized the advanced 90 nm CCD-in-CMOS process to manufacture test chips based on the simulation process conditions. The measured results generally matched the 3D simulation analysis. In the end of Section 5, drawing upon the observed 3D effects in the simulations, this project further optimized the full well performance and operational range of the ABG for the pixels. The optimized pixels showed significant improvements in full well performance while maintaining high charge transfer efficiency.

Table 3 shows the performance comparison between our work and other TDI imaging sensors based on the CCD-in-CMOS process. Based on the performance comparison with [1,14,25,26], much of the research on TDI image sensors has focused on the design of small pixels for large well capacity that can adapt to high line frequency operating conditions. From Table 3, compared with [25,26], it can be observed that after optimizing the FWC performance, our designed sensors still have a certain gap compared to the state of the art in terms of overall performance indicators (such as working frequency, FWC, and dark current). Therefore, further optimizing the performance of small pixels based on accurate 3D simulation models, making it suitable for high-speed and large-FWC applications, will be a key focus of future research work.

## Figures and Tables

**Table 1:** $V_{EFF}$ comparison between simulations and PCM test.

*V_EFF_*	3.5 μm Pixel	7.0 μm Pixel
2D simulation	1.1	1.12
3D simulation	1.0	1.11
PCM test	1.02	1.12

**Table 2:** FWC comparison between simulation and test chip measurement.

FWC	3.5 μm Pixel	7.0 μm Pixel
2D simulation	1.1	5.4
3D simulation	1.0	5.9
Test chip measurement	1.2	6.4

**Table 3:** Performance comparison between our work and other TDI imaging sensors.

Parameters	This Work	This Work (After *V_EFF_* Optimization)	[1]	[14]	[26]	[25]
Technology	90 nm	90 nm	N/A	130 nm	150 nm	N/A-
Pixel size	3.5 μm	3.5 μm	2 × 5 μm	5 μm	7.5 μm	12 × 3 μm
Working frequency	>5 kHz	>5 kHz	3 MHz	57 kHz	15 kHz	10 kHz
Full well	3 ke-	9.0 ke-	>60 ke-	20 ke-	92 ke-	58.6 ke-
Charge density per unit area	244 e-/μm^2^	732 e-/μm^2^	600 e-/μm^2^	800 e-/μm^2^	1635 e-/μm^2^	1627 e-/μm^2^
Conversion gain	62.6 μV/e-	62.6 μV/e-	13 μV/e-	23 μV/e-	13 μV/e-	22 μV/e-
QE	>80%	>80%	N/A	>70%	N/A	-N/A
CTE	>0.99998	0.99999	0.99999	0.99995	0.99999	0.999971
Dark current	13.7 nA/cm^2^	21.35 nA/cm^2^	<4 nA/cm^2^	12 nA/cm^2^	0.3 nA/cm^2^	0.28 nA/cm^2^
Signal-to-noise ratio	34.7 dB	39.5 dB	N/A	N/A	N/A	N/A
Anti-blooming capability	>50 × FWC	>50 × FWC	N/A	N/A	N/A	N/A

## Data Availability

The data are contained within the article.

## Abbreviations

TDI	Time Delay Integration
CCD-in-CMOS process	Charge-Coupled Device made via Complementary Metal Oxide Semiconductor process
FWC	Full Well Capacity
OFW	Optimum Full Well
CTI	Charge Transfer Inefficiency
CTE	Charge Transfer Efficiency
ADC	Analog-to-Digital Converter
MTF	Modulation Transfer Function
*V_EFF_*	Effective threshold voltage, maximum potential under barrier phase when the buried channel is pinned
*V_OFW_*	Optimum full well potential
*V_c_*	Gate voltage of collection phase in CCD
*V_C_surface_*	Potential at Si-SiO_2_ interface under collection phase
*V_C_max_*	Maximum potential in buried channel under collection phase
*V_b_*	Gate voltage of barrier phase in CCD
ABG	Anti-Blooming Gate
ABD	Anti-Blooming Drain
STI	Shallow Trench Isolation
TCAD	Technology Computer-Aided Design
JEFT	Junction Field-Effect Transistor
FS method	Floating Source method
PCM	Process Control Monitor
CVG	Conversion Gain
QE	Quantum Efficiency
DC	Dark Current
SNR	Signal-to-Noise Ratio
